# Invertebrate Gonadotropin-Releasing Hormone-Related Peptides and Their Receptors: An Update

**DOI:** 10.3389/fendo.2017.00217

**Published:** 2017-09-06

**Authors:** Tsubasa Sakai, Akira Shiraishi, Tsuyoshi Kawada, Shin Matsubara, Masato Aoyama, Honoo Satake

**Affiliations:** ^1^Bioorganic Research Institute, Suntory Foundation for Life Sciences, Kyoto, Japan; ^2^Faculty of Science, Department of Biological Sciences, Nara Women’s University, Nara, Japan

**Keywords:** gonadotropin-releasing hormone, adipokinetic hormone, corazoin, receptor, invertebrate

## Abstract

Gonadotropin-releasing hormones (GnRHs) play pivotal roles in reproductive functions via the hypothalamus, pituitary, and gonad axis, namely, HPG axis in vertebrates. GnRHs and their receptors (GnRHRs) are likely to be conserved in invertebrate deuterostomes and lophotrochozoans. All vertebrate and urochordate GnRHs are composed of 10 amino acids, whereas protostome, echinoderm, and amphioxus GnRH-like peptides are 11- or 12-residue peptide containing two amino acids after an N-terminal pyro-Glu. In urochordates, *Halocynthia roretzi* GnRH gene encodes two GnRH peptide sequences, whereas two GnRH genes encode three different GnRH peptides in *Ciona intestinalis*. These findings indicate the species-specific diversification of GnRHs. Intriguingly, the major signaling pathway for GnRHRs is intracellular Ca^2+^ mobilization in chordates, echinoderms, and protostomes, whereas *Ciona* GnRHRs (Ci-GnRHRs) are endowed with multiple GnRHergic cAMP production pathways in a ligand-selective manner. Moreover, the ligand-specific modulation of signal transduction via heterodimerization among Ci-GnRHR paralogs suggests the species-specific development of fine-tuning of gonadal functions in ascidians. Echinoderm GnRH-like peptides show high sequence differences compared to those of protostome counterparts, leading to the difficulty in classification of peptides and receptors. These findings also show both the diversity and conservation of GnRH signaling systems in invertebrates. The lack of the HPG axis in invertebrates indicates that biological functions of GnRHs are not release of gonadotropins in current invertebrates and common ancestors of vertebrates and invertebrates. To date, authentic or putative GnRHRs have been characterized from various echinoderms and protostomes as well as chordates and the mRNAs have been found to be distributed not only reproductive organs but also other tissues. Collectively, these findings further support the notion that invertebrate GnRHs have biological roles other than the regulation of reproductive functions. Moreover, recent molecular phylogenetic analysis suggests that adipokinetic hormone (AKH), corazonin (CRZ), and AKH/CRZ-related peptide (ACP) belong to the GnRH superfamily but has led to the different classifications of these peptides and receptors using different datasets including the number of sequences and structural domains. In this review, we provide current knowledge of, and perspectives in, molecular basis and evolutionary aspects of the GnRH, AKH, CRZ, and ACP.

## Introduction

Discovery of gonadotropin-releasing hormones (GnRHs) as a hypothalamic releasing factor for luteinizing hormone (LH) by Andrew V. Schally and Roger Guillemin in 1971 paved the way for investigation of basal endocrine reproductive systems (1, 2). This is also the origin of long and wide exploration of the GnRH kingdom. Over the past 20 years, GnRH and its related peptides have been identified in the central nervous system of not only non-mammalian vertebrates but also invertebrates such as ascidians, amphioxus, echinoderms, annelids, and mollusks (3–6). Invertebrates lack orthologs of gonadotropin hormones and pituitary glands, indicating that invertebrate GnRHs cannot serve as “gonadotropin-releasing hormones” in the hypothalamus, pituitary, and gonad axis (HPG axis) but rather function as neuropeptides that directly regulate target tissues. The expression of GnRH receptors (GnRHRs) in various tissues also supports non-hypothalamic functions of invertebrate GnRHs.

**Figure 1:**
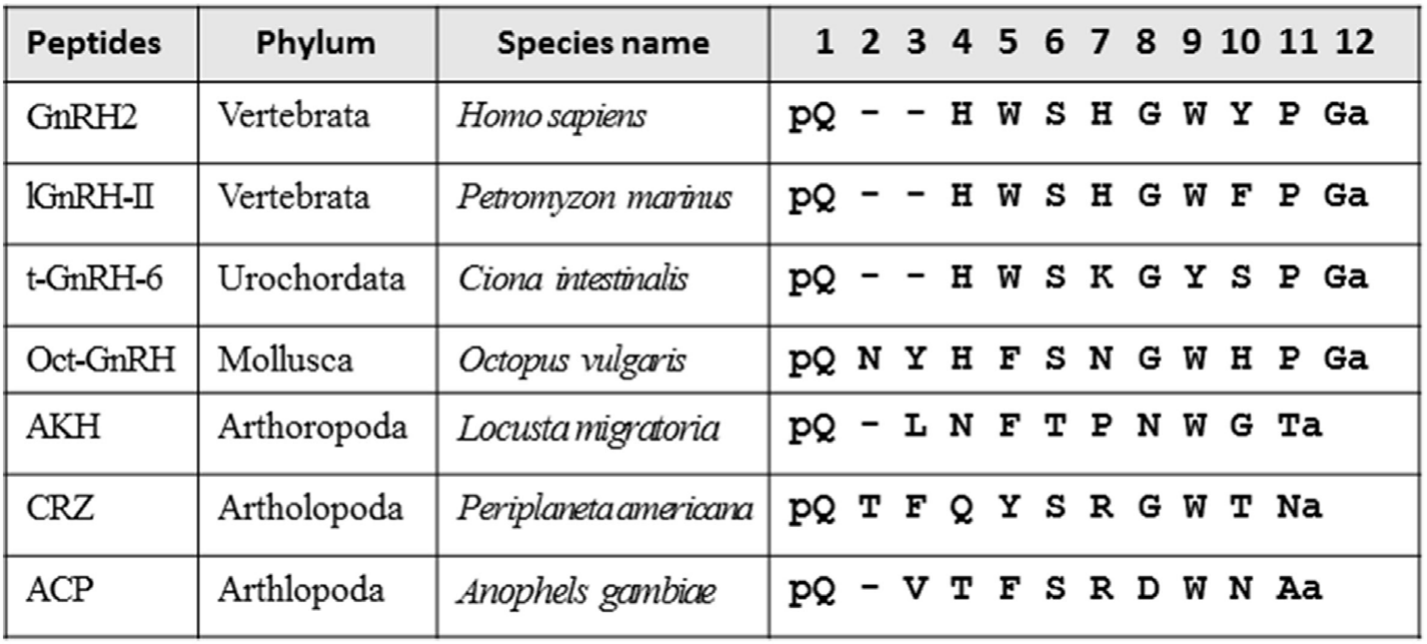
Amino acid sequence alignment of vertebrate gonadotropin-releasing hormones (GnRHs), urochordate GnRH, molluscan GnRH, adipokinetic hormone (AKH), corazonin (CRZ), and AKH/CRZ-related peptide (ACP).

**Figure 2:**
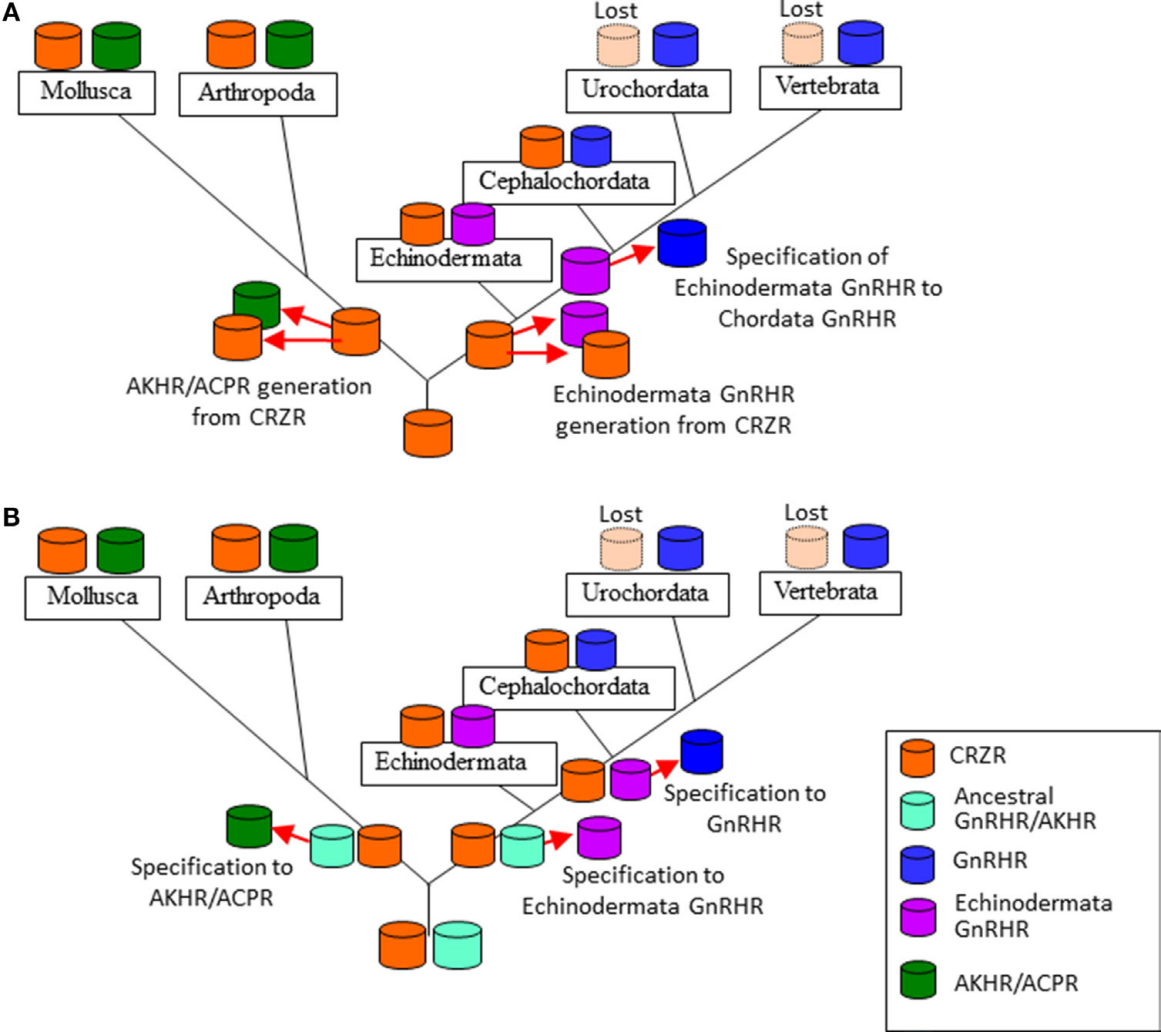
Two evolutionary scenarios of the formation of the gonadotropin-releasing hormone receptor (GnRHR) superfamily. **(A)** an ancestral corazonin receptor (CRZR), which has been conserved in the Echinodermata and Hemichordata, and the Annelida and the Mollusca, generated two lineages; (1) leading to CRZR and adipokinetic hormone receptor (AKHR), subsequently AKHR generated artholopod ACPR, (2) leading to GnRHR in the Chordata. CRZR was lost after evolution of the Urochordata in deuterostomes. **(B)** GnRHR and CRZR might have been arisen from an ancestral peptide receptor by gene duplication in a common ancestor of the Bilateria and a second gene duplication of GnRHR gave rise to the AKHR and ACPR in the Protostomia. CRZR has been preserved in all phyla except the Urochortada and the Vertebrata.

Various neuropeptides structurally related to GnRHs (Figure 1), such as adipokinetic hormone (AKH), corazonin (CRZ), and AKH/CRZ-related peptide (ACP), have also been identified in diverse invertebrates (3–11). As shown in Figure 1, these peptides share the N-terminal pyro-Glu residue and C-terminal amide and conserve Phe, Trp, or Tyr residue in position 3, Ser or Thr in position 4, and Trp or Tyr in position 7 with a vertebrate GnRH2 (pQHWSHGWYPGa). Furthermore, molecular phylogenetic and phylogenomic analyses of peptide genes have led to the presumption that GnRH, AKH, CRZ, and ACP originated from common ancestors of the Bilateria (3, 7–9), whereas the four peptides have been shown to exhibit distinct physiological functions including activation of lipid-mobilization by AKHs, stimulation of heart rate by CRZs (10), and down-regulation of oocyte proliferation and elevation of total hemolymph lipids by ACP (11) in arthropods. The cognate receptors for these peptides have also been identified in a wide invertebrate species, revealing that all of these receptors belong to the Class A G protein-coupled receptor (GPCR) family (5, 8, 12). Furthermore, sequence comparison and molecular phylogenetic analysis of these receptors have proposed several evolutionary scenarios for hundred million years (Figure 2), leading to the presumption that GnRH, AKH, CRZ, and ACP and their receptors constitute a superfamily (3, 5, 9, 13).

In this review, we provide basic and the latest knowledge regarding primary sequences, signal transductions, biological activities of GnRH, AKH, CRZ, and ACP and their receptors, and an overview of molecular evolution of these peptides and receptors.

### Gonadotropin-Releasing Hormones

#### Vertebrate GnRHs

Gonadotropin-releasing hormones are composed of 10 amino acids with consensus sequences of pyro-Glu^1^-His^2^-Trp^3^-Ser^4^ and Pro^9^-Gly^10^-amide and play pivotal roles in reproduction as releasing factor of gonadotropins in vertebrates (4). As shown in Table 1, two types of GnRHs (GnRH1 or Type 1 GnRH and GnRH2 or Type 2 GnRH) have been characterized in most vertebrates, whereas the third subtype was found in teleost and lamprey (14–16). Molecular phylogenetic tree and phylogenetic genomic analyses suggest that these subtypes have been generated by gene duplications within the species (15, 16). In other words, teleost GnRH3 and lamprey GnRH-III are specific paralogs to the respective species.

**Table 1:** Amino acid sequences of gonadotropin-releasing hormones (GnRHs).

**GnRH**			
**Deuterostome**			
**Vertebrate**			
Human Guinea pig Trout Lamprey	Homo sapiens Cavia porcellus Oncorhynchus mykiss Petromyzon marinus	GnRH1 GnRH2 GnRH1 GnRH3 l-GnRH-I l-GnRH-II l-GnRH-III	pQ--HWSYGLRPGa pQ--HWSHGWYPGa pQ--HWSYGVRPGa pQ--HWSYGWLPGa pQ--HYSLEWKPGa pQ--HWSHGWFPGa pQ--HWSHDWKPGa
**Invertebrates chordate**			
**Urochodate**			
Tunicate	Chelyosoma productum Ciona intestinalis Ciona savignyi Ciona intestinalis Halocynthia roretzi	t-GnRH-1 t-GnRH-2 t-GnRH-3 t-GnRH-4 t-GnRH-5 t-GnRH-6 t-GnRH-7 t-GnRH-8 t-GnRH-9 Ci-GnRH-X t-GnRH-10 t-GnRH-11	pQ--HWSYGLRPGa pQ--HWSLCHAPGa pQ--HWSYEFMPGa pQ--HWSNQLTPGa pQ--HWSYEYMPGa pQ--HWSKGYSPGa pQ--HWSYALSPGa pQ--HWSLALSPGa pQ--HWSNKLAPGa pQ--HWSNWWIPGAPGYNGa pQ--HWSYGFSPGa pQ--HWSYGFLPGa
**Cephalochordate**			
Amphioxus	*Branchiostoma floridae*	Amph.GnRHv Amph.GnRH	pQE-HWQYGHWYa pQILCARAFTYTHTWa
**Echinodermata**			
Sea urchin Starfish	Strongylocentrotus purpuratus Asterias rubens	Sp-GnRHP Ar-GnRH	pQVHHRFSGWRPGa pQIHYKNPGWGPGa
**Protostomes**			
**Mollusks and annelid**			
Octopus Cuttlefish Swordtip squid Oyster Yesso scallop Sea hare Owl limpet Marine worm Leech	Octopus vulgaris Sepia officinalis Loligo edulis Crassostrea gigas Patinopecten yessoensis Aplysia californica Lottia gigantean Capitella teleta Helobdella robusta	Oct-GnRH Oct-GnRH Oct-GnRH Cg-GnRH Py-GnRH Ap-GnRH Lg-GnRH Ca-GnRH Hr-GnRH	pQNYHFSNGWHPGa pQNYHFSNGWHPGa pQNYHFSNGWHPGa pQNYHFSNGWQPa pQNFHYSNGWQPa pQNYHFSNGWYAa pQHYHFSNGWKSa pQAYHFSHGWFPa pQSIHFSRSWQPa

*The N-terminal pyroglutamic acid and C-terminal amide are shown by “pQ” and “a,” respectively*.

#### Urochordate GnRHs

To date, 12 GnRH peptides have been identified in ascidians (Table 1). t-GnRH-1 and -2 were originally identified within the neural extract of an ascidian, *Chelyosoma productum* (17). Subsequently, ascidian GnRHs were isolated from other ascidians, *Ciona intestinalis* and *Ciona savignyi* (18). The former ascidian produces t-GnRH-3 to -8, and the latter generates t-GnRH-5 to -9 (18). In *Halocynthia roretzi*, t-GnRH-10 and -11 were characterized (19). All of these ascidian GnRHs conserve the consensus sequences of pyro-Glu^1^-His^2^-Trp^3^-Ser^4^ and Pro^9^-Gly^10^-amide of vertebrate GnRHs. Furthermore, a unique GnRH-related peptide, Ci-GnRH-X, was isolated from the neural tissue of *C. intestinalis* and was found to be composed of 16 amino acids harboring the consensus sequence of pyro-Glu^1^-His^2^-Trp^3^-Ser^4^ and Pro^9^-Gly^10^ and C-terminal Gly-amide (4, 20). The striking feature of ascidian GnRHs is multicopies of GnRH sequences in a single precursor, unlike vertebrate and non-ascidian invertebrate GnRH genes that encode a single GnRH sequence (4, 21). For instance, *ci-gnrh-1* encodes t-GnRH-3, -5, and -6, whereas t-GnRH-4, -7, and -8 sequences are found in another gene, *ci-gnrh-2* (18). Likewise, the *H. roretzi* GnRH gene encodes t-GnRH-10 and -11 (19). These findings indicate conservation and species-specific diversification of GnRHs in urochordates.

#### Cephalochordate and Echinoderm GnRH-Like Peptides

In the cephalochordate (amphioxus), *Branchiostoma floridae*, a GnRH-like peptide, Amph.GnRHv (pQEHWQYGHWYa, Table 1) was identified (12). Recently, GnRH-like peptides, SpGnRHP and ArGnRH (Table 1), were identified in the echinoderms, the sea urchin, *Strongylocentrotus purpuratus* (22) and the starfish, *Asterias rubens* (8), respectively. Unlike vertebrate and ascidian GnRHs, SpGnRHP and ArGnRH are 12-residue peptides containing a Val^2^-His^3^ or Ile^2^-His^3^ sequence, respectively (Table 1). These peptides share several amino acids with urochordate and vertebrate GnRHs and protostome GnRH-like peptides, including the N-terminal pGlu, His^4^ (corresponding His^2^ in chordate GnRHs), Gly^8^ (corresponding Gly^6^ in vertebrate GnRHs), Trp^9^ (corresponding Trp^7^ in vertebrate GnRHs), and C-terminal Pro-Gly-amide, whereas the GnRH N-terminal consensus motif displays quite low sequence homology (Table 1). Thus, categorization of the echinoderm peptides as the authentic GnRH family may remain to be concluded.

#### Protostome GnRH-Like Peptides

Over the past 15 years, GnRH-like peptides have been identified in protostomes including mollusks and annelids (4) (Table 1): an octopus, *Octopus vulgaris*; a cuttlefish, *Sepia officinalis*; a pacific oyster, *Crassostrea gigas*; a sea hare, *Aplysia californica*; a marine worm, *Capitella teleta*; a leech, *Helobdella robusta*; a scallop, *Patinopecten yessoensis*. Noteworthily, two-amino acid insertion after position 1 is found in all protostome GnRH-like peptides (Table 1). Collectively, these GnRH sequences indicate that 10-amino acid sequence length is conserved within ascidians and vertebrates, whereas protostome and non-chordate invertebrate GnRHs are featured by 2-amino acid insertion. In other words, ancestral GnRHs might have harbored such two amino acids after pyro-Glu, which might have been lost during the chordate evolutionary process.

The C-terminal Pro-Gly-amide of ascidian and vertebrate GnRHs is found in oct-GnRH of cephalopods and echinoderms but not in GnRH-like peptides of gastropods, bivalves, and annelids (Table 1), suggesting that cephalopods and echinoderms might have conserved the C-terminal Pro-Gly during their evolutionary processes. Furthermore, all known protostome GnRH-like peptides and SP-GnRHP share the Ser in position 6 or 7, while the Gly^8^-Trp^9^ sequence is conserved in cephalopod GnRH, echinoderm GnRHs, and l-GnRH-II but not in ascidian GnRHs and other vertebrate GnRHs except l-GnRH-II (Table 1). Additionally, substitution of Trp^3^ in the N-terminal consensus motif with Phe was found in most protostome GnRHs (Table 1). Altogether, these sequences led to the presumption that the ancestral GnRHs might have been composed of pQ-H(F/W)S-GW-PGa or pQ-H(F/W)S-GW-a, and thereafter, chordate GnRHs might have diverged via various substitution and deletion of the two N-terminal amino acids in the evolutionary process of each species.

#### Vertebrate GnRHRs

Gonadotropin-releasing hormone receptors belong to the Class A GPCR family (4, 14). In most vertebrates, two or three molecular forms of GnRHRs are present (14). Molecular phylogenetic analyses have provided evidence that vertebrate GnRHRs are classified into three groups, type-I, -II, and -III. The type-I GnRHRs were characterized from a wide range of vertebrate species such as teleost, amphibians, reptiles, birds, and mammals (23). Mammalian type-I GnRHRs completely lack the C-terminal tail region, which is present in its non-mammalian receptors (14). The type-II *gnrhr* gene is found in the genome of amphibians, reptiles, aves, and mammals (23). Most mammalian type-II *gnrhr* is non-functional due to the deletion of functional domains or interruption of full-length translation by the presence of a stop codon. In contrast, type-II GnRHRs of several monkeys, pigs, and other non-mammalian vertebrates were shown to be functional (23). Type-I GnRHRs show high affinity for both GnRH1 and 2, whereas type-II GnRHRs are specifically responsive to GnRH2 (14). Type-III GnRHRs were identified in non-mammalian vertebrates (24). In chicken, type-III GnRHR exhibits a 35-fold higher affinity for GnRH2 than for GnRH1 (24). GnRHRs are in general coupled with Gq protein and activate a typical phospholipase C (PLC)–inositol triphosphate (IP)–intracellular calcium mobilization signaling cascade, occasionally leading to phosphorylation of mitogen-activated protein kinase (MAPK) including ERK1/2 (4, 25, 26), while some GnRHRs are also found to trigger or suppress cAMP production via coupling with Gs or Gi protein (21, 25–28).

#### Invertebrate GnRHRs

##### Ascidian GnRHRs

In *C. intestinalis*, four GnRHRs, *Ciona* GnRHR (Ci-GnRHR)-1 to -4, have been identified and shown to regulate exceptionally complicated signaling pathways involving ligand-receptor selectivity, coupling with multiple G-protein subtypes, and receptor heterodimerization (Table 2).

**Table 2:** Characteristics of ascidian gonadotropin-releasing hormone (GnRH) receptors.

Receptor	Preferable ligands	G proteins	Signaling pathway	Effect by *Ciona* GnRHR (Ci-GnRH)-X	Effect by heterodimerization with R-4
Ci-GnRHR-1	t-GnRH-6	Gq, Gs	Ca^2+^, cAMP	Moderate inhibition	Potentiation of Ca^2+^ signaling
Ci-GnRHR-2	t-GnRH-7, -8, -6	Gs	cAMP	No effect	Decreasing cAMP production
Ci-GnRHR-3	t-GnRH-3, -5	Gs	cAMP	Moderate inhibition	None
Ci-GnRHR-4	No ligand	None	None	None	–

*Ciona* GnRHR-1, -2, and -3 sequences were found to harbor a long C-terminal tail, whereas a short tail is present in the C-terminus of Ci-GnRHR-4 (27). Ci-GnRHR mRNAs are distributed in the neural complex, heart, intestine, endostyle, branchia sac, and ovary, although biological roles of GnRHs largely remain unclear (26–28). Notably, the elevation of intracellular calcium, which is a typical response of GnRHR activation, was observed only in the t-GnRH-6 and Ci-GnRHR-1 pair (27). t-GnRH-6 also induces cAMP production via Ci-GnRHR-1 (27). Ci-GnRHR-2 exclusively stimulates cAMP production in response to t-GnRH-7, -8, and -6 in this order of potency (27). Ci-GnRHR-3 triggers cAMP production in the presence of t-GnRH-3 and -5 to a similar extent in a ligand-specific fashion. Ci-GnRHR-4 exhibited neither elevation of intracellular calcium nor production of cAMP (27). Induction of intracellular mobilization only by t-GnRH-6 and Ci-GnRHR-1 pair is attributed to the conservation of Gly^6^ essential for adoption of the tertiary structure for coupling with Gq (14) exclusively in t-GnRH-6 (Table 1). Such signaling profiles indicate that a major *Ciona* GnRH signaling is a cAMP production. Additionally, *Ciona* 16-amino acid GnRH-structurally related peptide, Ci-GnRH-X, was shown to exhibit moderately inhibit activation of Ci-GnRHR-1 and -3 (20). Also of particular interest in Ci-GnRHR signaling is that Ci-GnRHR-4 heterodimerizes with Ci-GnRHR-1 and then potentiates the elevation of intracellular calcium via both calcium-dependent and -independent protein kinase C subtypes and ERK phosphorylation in a ligand-selective fashion (26). Ci-GnRHR-4 was also found to heterodimerize with Ci-GnRHR-2 (28). The Ci-GnRHR-2/-4 heterodimer decreased cAMP production by 50% in a non-ligand selective manner by shifting of activation from Gs protein to Gi protein by Ci-GnRHR-2, compared to the Ci-GnRHR-2 monomer/homodimer (28). These findings verify that Ci-GnRHR-4 serves as a protomer of GPCR heterodimers rather than a ligand-binding receptor (4, 21, 29). In addition, molecular phylogenetic analysis demonstrated that Ci-GnRHRs are included in vertebrate GnRHR clades but form an independent cluster in chordate GnRHRs, suggesting that these receptors have evolved within the *Ciona* species (4, 21, 27, 29, 30). Collectively, these findings indicate ascidian-specific molecular and functional diversity of ascidian GnRH signaling systems.

##### Amphioxus GnRHRs

Four amphioxus receptors have been identified in the amphioxus, *B. floridae*. Amphioxus GnRHR-1 and -2 were activated only by vertebrate GnRHs but not by Amph.GnRHv, a putative *B. floridae* endogenous GnRH-like peptide that displays the highest sequence similarity to other species GnRHs (Table 1), whereas GnRHR-3 was activated exclusively by another amphioxus GnRH- and CRZ-like peptide (Table 2), oct-GnRH, and AKH at physiological concentrations (12, 31), indicating that amphioxus GnRHR-3 exhibits extensive ligand selectivity for GnRH superfamily peptides. Unlike Ci-GnRHRs, *B. floridae* GnRHR-1 to -3 were shown to stimulate only intracellular IP accumulation (12). In contrast, no ligands induced IP accumulation or cAMP stimulation via amphioxus GnRHR-4 (12, 31). It should be noted that the Amph.GnRHv failed to activate any of the four GnRHRs (12). Molecular phylogenetic analysis demonstrated that amphioxus GnRHR-1 and -2 are included in the vertebrate GnRHR clade, while amphioxus GnRHR-3 and -4 are likely to belong to the CRZR/GnRHR clade, as described later. Consequently, the authors presumed that the sequence of the neuropeptide might reflect ancestral sequence of CRZ/GnRH or the transition state between CRZ and GnRH (12). Moreover, of keen interest is the identification of authentic (endogenous) ligands for amphioxus GnRHR-1 and -2. Thus, the elucidation of authentic amphioxus GnRH–receptor pairs requires further investigation. Such difficulty may be attributed to some mismatch between amphioxus GnRHRs and cultured cells employed for heterologous functional analysis because of unsuccessful translation of the receptor mRNA or degradation of the receptor protein in heterologous expression systems (32).

##### Echinoderm GnRHRs

Tian et al. (8) demonstrated that Ar-GnRH (Table 1) specifically activated intracellular Ca^2+^ mobilization of a cognate receptor, ArGnRHR in the starfish, *A. rubens*. Four GnRH/CRZ-type receptors have also been identified in the sea urchin, *S. purpuratus* using *in silico* screening (22). However, no functional analysis of these receptors has been reported. Additionally, these echinoderm receptors are included in the invertebrate CRZ/GnRHR clade (12, 31).

##### Protostome GnRHRs

The first protostome GnRHR was identified in an octopus, *O. vulgaris*. The octopus GnRHR, oct-GnRHR, activates intracellular Ca^2+^ mobilization by oct-GnRH but not vertebrate GnRHs (33). Notably, an oct-GnRH synthetic analog with Asn^2^-Tyr^3^ deletion abolished the ability to activate the Ca^2+^ pathway via oct-GnRHR, whereas a chicken GnRH-II synthetic analog with an Asn-Tyr insertion after position 1 exhibited weak activation (33). These findings verify that Asn^2^-Tyr^3^ is required for the activation of oct-GnRHR, suggesting that the two amino acids after position 1 in non-chordate GnRHs are responsible for activating the protostomian GnRHR. *Oct-gnrhr* is expressed in the central nervous system, digestive tissues, aorta, heart, salivary gland, branchia, radula retractor muscle, egg, and genital organs in the common octopus (33). In another mollusk, gastropod (a sea hare) *A. californica* GnRHR, ap-GnRHR, was also cloned and was found to be expressed in the abdominal, cerebral, and buccal ganglia of the central nervous system and a few peripheral tissues including the chemosensory organ, small hermaphroditic duct, and ovotestis (13). ap-GnRH was shown to increase the IP accumulation but not cAMP production in ap-GnRHR-expressing *Drosophila* S2 cells in a ligand-specific manner (13). Phylogenetic analysis suggests that ap-GnRHR is clustered with several molluscan GnRHRs including oct-GnRHR, amphioxus GnRHR-3 and -4, and multiple insect CRZRs (13).

##### Biological Functions

In vertebrates, GnRH is synthesized in the hypothalamus, transported to the pituitary and triggers release of follicle-stimulating hormone (FSH) and LH from the pituitary, eventually regulating reproductive functions via the HPG axis. GnRH also serves as a peripheral bioactive peptide including induction of the synthesis and release of sex steroids in vertebrate reproductive tissues (14). The HPG axis-directed endocrine systems were acquired during the vertebrate evolutionary process, and thus, invertebrate GnRHs are likely to have prototypic or species-specific biological roles.

In ascidians, GnRHs were found to increase water flow and then induce the release of eggs and sperm by injection into the gonaducts, ovary, stomach, and posterior body cavity of *C. intestinalis* (18, 34). All four *Ci-gnrhr* genes were shown to be expressed in the brain of the larva of *C. intestinalis* (30). *Ci-gnrhr-1* and *-2* genes are expressed in muscle cells, while *Ci-gnrhr-3* gene is expressed in notochord cells in the larval tail, which is rapidly resorbed during metamorphosis (30). Intriguingly, Kamiya et al. (35) demonstrated that tGnRH-3 and -5 suppressed the growth of adult organs by arresting cell cycle progression and the promotion of tail absorption. These results indicate that t-GnRHs play a pivotal role in the development and/or metamorphosis.

oct-GnRH induced contraction of the oviduct (36) and releases sex steroids, including testosterone-, progesterone-, and 17β-Estradiol-like steroids from the follicle and spermatozoa in octopus (33). In another mollusk, the yesso scallop (*Patinopecten yessoesis*), py-GnRH induces testicular cell proliferation (37). These findings suggest that molluscan GnRHs directly activate the gonadal organs as a bioactive peptide. In contrast, injection of the cognate ap-GnRH into sexually mature and immature sea hares exhibited no effects on ovotestis mass, reproductive tract mass, egg-laying, or penile eversion, altering oocyte growth and egg-laying hormone accumulation and secretion (38). Instead, ap-GnRH exerted stimulation of the parapodial opening, inhibition of feeding, and promotion of substrate attachment (38). These findings, combined with distribution of GnRHR mRNAs in various tissues, suggest that invertebrate GnRHs regulate not only reproductive responses but also other various biological behaviors. Indeed, oct-GnRH induced contraction of the radula retractor muscle expressing *oct-gnrhr* (33).

### Adipokinetic Hormones

Adipokinetic hormone was originally identified in the migratory locust, *Locusta migratoria* as a lipid mobilizing factor (39). To date, AKHs have been isolated from insects, mollusks, and nematode (Figure 1; Table 3). AKHs are composed of 8–10 residues, harboring pGlu in position 1, an aliphatic or aromatic amino acid residue at position 2, Phe-Ser, Phe-Thr, or Tyr-Ser residues at positions 4 and 5, Trp at position 8, and Trp-amide, Trp-Gly-amide, or Trp-Gly-X-amide (where “X” is variable) at the C terminus (5). Li et al. have proposed to classify these peptides in the Protostomia as follows: authentic AKHs that fulfill the above hallmarks, AKH-like peptides that 10 amino acid residues have Trp-X-Gly-amide or Trp-X-Pro-amide at the C terminus, and proto-AKHs that are longer than 10 amino acid residues but have only 2–4 of the AKH hallmarks (40).

**Table 3:** Amino acid sequences of adipokinetic hormones (AKHs).

**AKHs**			
**Protostome**			
**Mollusks**			
Oyster Owl limpet Sea hare	Crassostrea gigas Lottia gigantea Aplysia californica	Cg-AKH Lg-AKH Ap-AKH	pQ-VSFSTNWGSa pQ-IHFSPTWGSa pQ-IHFSPDWGTa
**Arthropod**			
Centipede Fruit fly Silk worm Locust	Strigmaia maritima Drosophila melanogaster Bombix mori Locusta migratoria	Smar-AKH Dm-AKH Bm-AKH1 Lm-AKH3	pQ-INFSPGWGQa pQ-LTFSPDWa pQ-LTFTSSWGa pQ-LNFTPWWa
**Nematode**			
Nematode	*Caenorhabditis elegans*	Ce-AKH	pQ-MTFTDQWT

*The N-terminal pyroglutamic acid and C-terminal amide are shown by “pQ” and “a,” respectively*.

#### AKH Receptors

Adipokinetic hormone receptors belong to the Class A GPCR family identified in protostomes. Zhu et al. demonstrated that AKH activates both cAMP accumulation and Ca^2+^ mobilization via AKHR of the silkworm moth, *Bombix moli* (41). Recently, Li et al. demonstrated that AKHR of the oyster *Crassostrea gigas* was activated by oyster AKH at physiological concentrations (40). Moreover, Nagasawa et al. detected expression of *Py-AKHR* mRNAs in the nerve ganglia, lip, foot, CPG, mantle, testis, and ovary in Yesso scallop, *Patinopecten yessoensis*. The differential expression profile of *Py-AKHR* mRNA in the gonad during gonadal maturation stages suggests their reproductive function (42).

#### Biological Functions

Adipokinetic hormones have so far been shown to stimulate the fat body, resulting in lipid and carbohydrate mobilization into the hemolymph in insects and crustaceans. Furthermore, a homolog of AKH in the northern shrimp, *Paudalus borealis*, red pigment concentrating hormone, influenced the concentration of pigment chromatophore, causing its body color change (43). Notably, AKHs also showed a reduction in oocyte protein and carbohydrate content in the crickets, *Gryllus bimaculatus*, and a reduction in vitellogenin of oocytes in *L. migratoria* (44), indicating a regulatory role for AKHs in insect reproduction. AKH-deficient flies displayed the opposite phenotype in which hemolymph trehalose levels decreased and storage lipid in the fat body accumulated (45). An AKH receptor-deficient strain showed a similar phenotype to AKH-deficient flies (46). In the cricket *G. bimaculatus*, AKH receptor knockdown by RNAi increased feeding frequency and reduced locomotor activity (47).

### Corazonins

Corazonins were originally characterized as 11-amino acid arthropod neuropeptides from the cockroach, *Periplaneta americana* (10). A striking feature is the highest conservation of sequence similarity of CRZs in the Arthropoda regardless of diverse functions throughout a variety of species (Table 4). [Arg^7^]-CRZ (pQTFQYSRGWTN-amide) is the most typical CRZ peptide, and only a few homologs such as [His^7^]-CRZ, [Gln^10^]-CRZ, and [His^4^-Gln^7^]-CRZ have been found in several insects (5). Recently, however, neuropeptides weakly similar to CRZ have been identified in starfish (HNTFTMGGQNRWKAG-amide), sea urchin (HNTFSFKGRSRYFP – amide), and acorn worm (pQPHFSLKDRYRWK-amide) (Table 4), and the starfish peptide was shown to be responsive to the cognate CRZ-type receptor, leading to the presumption that these peptides are invertebrate deuterostome CRZs as putative CRZ-type receptor ligands (8).

**Table 4:** Amino acid sequences of corazonins (CRZs).

**CRZ**			
**Hemichordate**			
Acorn worm	*Saccoglossus kowalevskii*	Sk-CRZ-like	pQPHFSLKDRYRWKPa
**Echinoderm**			
Sea urchin Starfish	Strongylocentrotus purpuratus Asterias rubens	Sp-CRZ-like Ar-CRZ-like	HNTFSFKGRSRYFPa HNTFTMGGQNRWKAGa
**Arthropod**			
Most arthropods Centipede Locust	– Strigmaia maritima Locusta migratoria	CRZs Smar-CRZ Lm-CRZ	pQTFQYSRGWTNa pQTFQYSKGWEPa pQTFQYSHGWTNa

*The N-terminal pyroglutamic acid and C-terminal amide are shown by “pQ” and “a,” respectively*.

#### CRZ Receptors (CRZRs)

**Figure 3:**
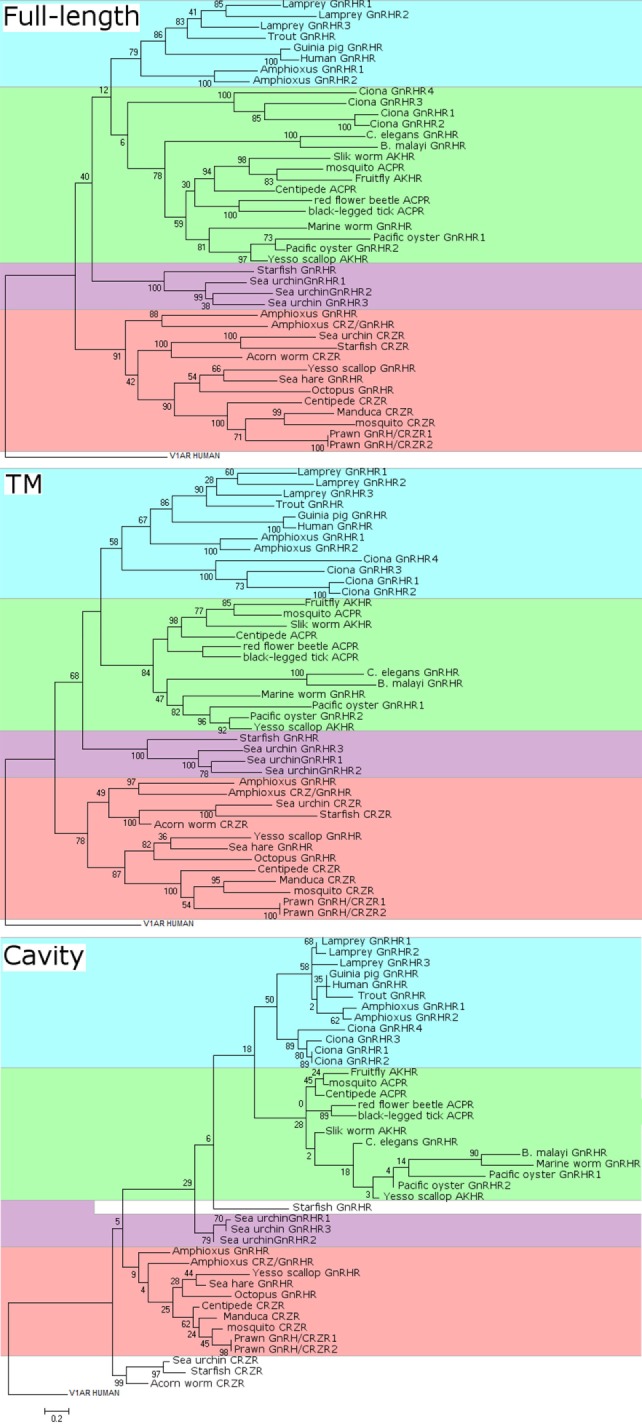
Molecular phylogenetic analysis of full-length (top), transmembrane (TM) domain (middle), and cavity (bottom) sequences of gonadotropin-releasing hormone receptors (GnRHRs), AKHRs, corazonin receptors (CRZRs), and ACPRs. The sequence alignments were constructed using MUSCLE in MEGA version 7 and GPCRalign (61) for full-length alignments and TM alignments, respectively. GPCRalign is a PSSM-based alignment algorism and output total 201-length gapless alignments corresponding to TM region. The output TM sequences are listed in Supplementary Material 1–7. The cavity amino acid positions in TM alignment were extracted according to previous report (60). The cavity sequences are listed in Supplementary Material 8. A phylogenic tree of GnRHRs was constructed by the maximum likelihood method based on the JTT matrix-based model. For full-length phylogenetic tree, all positions containing gaps and missing data were eliminated. The scale bar indicates the evolutionary distance of 0.2 amino acid substitutions per protein. The number at each branch node represents percentage given by 100 bootstrap replicates. Evolutionary analyses were conducted in MEGA version 7. The sequences used were as follows: human GnRHR (GNRHR_HUMAN); guinea pig GnRHR (GNRHR_CAVPO); marine worm GnRHR (R7U4C9_CAPTE); sea urchin GnRHR-1 (B2BF80_STRPU); sea urchin GnRHR-2 (B2BF81_STRPU); sea urchin GnRHR-3 (B2BF82_STRPU); tunicate GnRHR-1 (Q869J2_CIOIN); tunicate GnRHR-2 (Q869J1_CIOIN); tunicate GnRHR-3 (D2KZ68_CIOIN); tunicate GnRHR-4 (D2KZ69_CIOIN); trout GnRHR (Q9I986_ONCMY); lamprey GnRHR-1 (A9XCD3_PETMA); lamprey GnRHR-2 (A9XCD4_PETMA); lamprey GnRHR-3 (A9XCD5_PETMA); octopus GnRHR (GNRHR_OCTVU); amphioxus GnRHR-1b (A9XCD1_BRAFL); amphioxus GnRHR-2b (A9XCD2_BRAFL); amphioxus GnRHR-3 (C0IP22_BRAFL); amphioxus GnRHR-4 (C4N9P5_BRAFL); pacific oyster GnRHR-2 (B1GVI7_CRAGI); nematode GnRHR (O44731_CAEEL); sea hare GnRHR (Refseqid:AHE78444); filarial nematode worm GnRHR (A8PVQ9_BRUMA); starfish GnRHR (A0A1B0YGS0_ASTRU); yesso scallop GnRHR (Refseqid: BAX08608); pacific oyster AKHr1b (B1GVI4_CRAGI); fruit fly AKHR (Q71EB3_DROME); silk worm AKHR (Q8T6U9_BOMMO); yesso scallop AKHR (Refseqid: BAX08609); centipede ACPR (Refseqid: AFFK01020326); red flour beetle ACPR (D5FFV2_TRICA); black-legged tick ACPR (A0A0 × 7YC79_IXOSC); honeybee CRZR (B7ZKE3_APIME); tobacco hawk moth CRZR (Q6UJG5_MANSE); sea urchin CRZR (Refseqid: XP_011680711); starfish CRZR (A0A1B0YGT7_ASTRU); centipede CRZR (Refseqid: AFFK01019957); and acorn worm CRZR (Refseqid: XP_006820827).

Corazonin receptors are class A family GPCRs. The first CRZR was identified in *Drosophila melanogaster*, and then orthologous receptors have been cloned from moths, mosquitoes, honey bee, and other insects (48) (Figure 3). CRZR of the silkworm moth *Bombix mori* induces cAMP accumulation, Ca^2+^ mobilization, and ERK1/2 phosphorylation via the Gq- and Gs-coupled signaling pathways in response to CRZ (49). Various molecular phylogenetic analyses indicate that the annelid and molluscan GnRHRs are clustered with the CRZRs and the annelid and molluscan GnRHRs have been recognized as the members of CRZR/GnRHR clade (5, 12, 13). In the starfish, *Asterias rubens*, CRZ-like peptide (HNTFTMGGQNRWKAG-amide) was identified and also found to activate the cognate receptor (8). Likewise, GnRHR-type receptor was identified and found to be activated specifically by the cognate GnRH-like peptide (pQIHYKNPGWGPG-amide) in a ligand-specific manner (8). Collectively, these results suggest that echinoderms, at least *A. rubens*, may be endowed with the GnRH- and CRZ-directed signaling systems (8).

Notably, as stated earlier, *B. floridae* (amphioxus) GnRHR-3 and -4 are highly homologous to the protostome CRZR/GnRHR receptor family and GnRHR-3 was activated by the amphioxus GnRH-like peptide (pQILCARAFTYTHTW-amide), oct-GnRH (pQNYHFSNGWHPG-amide), and AKH (pQLTFTSSW-amide) at physiological concentrations, indicating that *B. floridae* GnRHR-3 exhibits extensive ligand selectivity for GnRH superfamily peptides. The CRZ/CRZR signaling system has been lost in urochordates, vertebrates, nematodes, and some insects (50).

#### Biological Functions

Corazonins have a number of physiological roles associated with control of heartbeat, ecdysis behavior initiation, and cuticle coloration in the Artholopoda (5, 48). Recently, its regulatory functions on insulin producing cells in the brain of *D. melanogaster* (51) and on larval–pupal transition and pupariation behavior have been found in the fruit fly, *Bactrocera dorsalis* (52). Intriguingly, CRZs also show reproductive activities in invertebrates. In male flies, CRZs act on its receptor in a small cluster of posterior serotoninergic neurons to control activity of the accessory glands and sperm ejaculation during mating (53). In the giant freshwater prawn *Macrobrachium rosenbergii*, CRZs inhibit testicular development and spermatogenesis and androgenic gland secretion (54). Ablation of CRZ-GAL4 neurons increased locomotion and dopamine level in male files, *D. melanogaster*. Furthermore, silencing of CRZR-GAL4 neurons in male flies elicits infertility and blocks sperm and seminal fluid ejaculation (53). In *B. mori*, dsRNA-mediated knockdown of BmCrzR indicated a role of CRZ signaling in the regulation of silkworm growth and silk production (49).

### AKH/CRZ-Related Peptides

Adipokinetic hormone/CRZ-related peptide is a10–11-amino acid arthropod peptide originally identified from the malaria mosquito, *Anopheles gambiae* (55). In contrast to AKHs, sequences of ACPs, in particular, the N-terminal sequence “QXTFSRXW” (where “X” is variable) and C-terminal amidation, are well conserved in arthropods (Table 5), which is reminiscent of an intermediate between AKHs and CRZs (5).

**Table 5:** Amino acid sequences of adipokinetic hormone/corazonin-related peptides (ACPs).

**ACP**			
**Protostome**			
**Arthropod**			
Mosquito Kissing bug Flour beetle Centipede Prawn	Anopheles gambiae Rhodnius prolixus Tribolium castaneum Strigmaia maritima Macrobrachium rosenbergii	Agam-ACP Rhopr-ACP Tc-ACP Smar-ACP Mro-ACP	pQ-VTFSRDWNAa pQ-VTFSRDWNAa pQ-VTFSRDWNPa pQ-VTFSRDWTPAs pQ-ITFSRSWVPQa

*The N-terminal pyroglutamic acid and C-terminal amide are shown by “pQ” and “a,” respectively*.

#### ACP Receptors

Adipokinetic hormone/CRZ-related peptide receptors are Class A family GPCRs identified only in insects (Figure 3). Hansen et al. showed that the *A. gambiae* ACP receptor transfected into mammalian cells stably expressing the human G-protein G16, a universal G protein adapter, was activated specifically by the cognate ligand (55). Zandawala et al. characterized three splice variants encoding ACP receptors in the kissing bug *Rhodnius prolixus*; Rhopr-ACPR-A has only five transmembrane (TM) domains, and Rhopr-ACPR-B and C have seven TM domains. All Rhopr-ACPR-A, -B, and -C were activated by Rhopr-ACP but neither Rhopr-AKH nor Rhopr-CRZ with different sensitivities on mammalian cells stably expressing the G-protein G16, whereas Rhopr-ACPR-B and -C indicated coupling with Gq when expressed in CHO-K1-aeq cells (56).

#### Biological Functions

To date, the ACP signaling system has been found only in arthropods and its major biological roles are still unclear. However, recently, ACP was shown to decrease germ cell proliferation and increases in total hemolymph lipids were found by administration of the peptide in female prawn, *M. rosenbergii* (11). The expression of *MroACP* mRNA in the eyestalk, central nervous system, thoracic ganglia, and *MroACPR* mRNA in the neural tissues and the ovary throughout different stages of ovarian maturation indicated a neuronal regulation of ACP signaling in reproduction (11).

## Proposed Evolutionary Scenarios of GnRH, AKH, CRZ, ACP, and Their Receptors

Based on the aforementioned sequence homology and molecular phylogeny, several studies suggested that GnRH, AKH, CRZ, and ACP constitute a superfamily and originated from a common ancestor (57, 58). However, marked sequence diversity in GnRH, AKH, CRZ, and ACP has led to difficulty in accurate or conclusive classification. For example, Lindemans et al. suggested that GnRH signaling might have been arisen before the divergence of protostomes and deuterostomes on the basis of the presence of the AKH-GnRH signaling system in the nematode *Caenorhabditis elegans* and its biological function in the egg-laying behavior (59). However, molecular phylogenetic analysis led to another presumption that the *C. elegans* AKH-GnRH-like peptide and its receptor belong to the authentic AKH system (5, 50, 59).

As described early, authentic GnRHRs are conserved at least in the Cephalochordata, Urochordata, and the Vertebrata. AKHRs have been identified in the Mollusca, Annelida, and Arthropoda, while ACPRs have been found only in the Arthropoda. Authentic or putative CRZRs are present in all invertebrates except the Urochordata. Molecular phylogenetic analysis (3, 5, 8, 13, 50) has thus far provided two scenarios of their evolutionary processes. The first one is that an ancestral CRZR, which has been conserved in the Ambulacraria (the Echinodermata and the Hemichordata) and the Lophotrochozoa (the Annelida and the Mollusca), generated two lineages: (1) leading to CRZR and AKHR, subsequently AKHR generated artholopod ACPR in the Ecdysozoa and (2) leading to GnRH in the Chordata. CRZR was lost during the evolution of the Urochordata and Vertebrata in deuterostomes (Figure 2A). The second one is that GnRHR and CRZR might have been arisen via gene duplication in a common ancestor of the Bilateria, and a second gene duplication of GnRHR might have generated AKHR and ACPR during the divergence of the Lophotrochozoa and Ecdysozoa (the Arthropoda and the Nematoda). CRZR has been conserved in all phyla except the Urochordata and Vertebrata (Figure 2B). Notably, these receptors were categorized as different clusters by respective research groups, e.g., GnRHR/AKHR/ACPR and CRZ (8); GnRHR, AKHR/ACPR, and CRZR (13); GnRHR, AKHR/ACPR, and CRZR/protostome GnRHR (3); and GnRHR, AKHR, ACPR, CRZR/protostome GnRHR, and CRZR (5). Furthermore, based on the results of phylogenomic analyses with 36 whole genome sequences and no functional connection of protostome GnRH signaling system to the releasing of gonadotropins because of the lack of the HPG axis in protostomes, Plachetzki et al. classified protostome GnRHs as CRZ-like (or ACP/AKH-like) peptides and categorized the receptors of GnRH superfamily as GnRHRs and CRZR (or ACPR/AKHR) (9).

Such data are mainly attributed to difference in the number, length, and domain of sequences employed for molecular phylogenetic analysis. Figure 3 shows our reanalysis of the molecular phylogeny of full-length, TM domain, and ligand-binding cavity (60) sequences of 42 receptors including GnRHRs, AKHRs, CRZRs, and ACPRs. Full-length sequences of these receptors were aligned with CLUSTALW using BLOSUM62 substitution matrix. Amino acid sequences of the TM and the cavity were individually aligned with GPCRalign (61). All of molecular phylogenetic tree analyses of the full-length sequences (Figure 3) demonstrate that these receptors are classified into the following four major clusters: (1) vertebrate GnRHRs and amphioxus GnRHR-1 and -2 (highlighted in blue), (2) invertebrate GnRHRs/AKHRs/ACPRs including the urochordate GnRHRs, Ci-GnRHRs (highlighted in green), (3) echinoderm GnRHRs (highlighted in purple), and (4) protostome GnRHRs/amphioxus GnRHR-3 and 4/CRZRs (highlighted in orange). Of note, molecular phylogenetic analyses of the TM (Figure 3, middle) and cavity (Figure 3, bottom) sequences of these receptors indicate that Ci-GnRHRs and amphioxus GnRHR-1 and -2 are included in the clade of vertebrate GnRHRs, although many bootstraps in the molecular phylogenetic tree of the cavity are very low due to much smaller information of cavity sequences (30–40 amino acids) than that of full-length and TM, suggesting extremely weak evolutionary correlations. In contrast, Ci-GnRHRs are included in the AKHR/ACPR cluster in a molecular phylogenetic tree of the full-length sequences (Figure 3, top). Moreover, the molecular phylogenetic trees of the TM and the cavity regions indicate that echinoderm “GnRHRs” form a monophyletic clade and display closer homology to the CRZR family than the GnRHR family. This molecular phylogenetic tree is consistent with species-specific sequences of echinoderm GnRH-like peptides (Table 1), suggesting species-specific diversification of echinoderm GnRH and GnRHR lineages. In combination, conservation of partial consensus motifs and molecular phylogenetic analyses are not sufficient for substantiating the evolutionary process of the “GnRH/AKH/CRZ/ACP superfamily,” which may mislead us to an incorrect conclusion.

## Conclusion and Perspetives

In the Vertebrata, GnRHs play pivotal roles in reproductive function as a releasing factor for gonadotropin in the HPG axis and a neuropeptide that directly regulate target tissues. In contrast, reproductive functions of invertebrate GnRHs have not been demonstrated. Instead, there has been a growing body of reports of reproductive functions of invertebrate GnRH-related peptides, AKH, CRZ, and ACP. These findings suggest that, if GnRH, AKH, CRZ, and ACP constitute a superfamily, the superfamily peptides might have been endowed with both common and species-specific reproductive functions as well as other physiological functions. In this regard, of particular interest are biological roles of GnRHs or GnRH-like peptides in protostomes, echinoderms, cephalochordates, and urochordates, which lack the HPG axis.

Gonadotropin-releasing hormone, AKH, CRZ, and ACP bear approximately 10 amino acids, and the respective “consensus motifs” are frequently diverged among species. Furthermore, the nested clusters (Figure 3) within GnRHRs, AKHRs, and CRZRs in molecular phylogenetic trees of the TM and the cavity imply that a small number of amino acid substitutions in these regions can change their ligand selectivity. Therefore, only standard homology-based analysis may lead to insufficient data for understanding the evolutionary process of these peptides and receptors. Consequently, integration of multiple molecular phylogenetic analyses of much more sequence information of these peptides and receptors in other invertebrates with biological roles of these signaling systems in various invertebrate species will enable us to elucidate their biological significance and true evolutionary processes.

## Author Contributions

TS and HS conducted manuscript preparation. TS, TK, SM, MA and HS investigated background literatures. TS, AS and HS wrote manuscripts. AS and HS analyzed data.

## Conflict of Interest Statement

The authors declare that the research was conducted in the absence of any commercial or financial relationships that could be construed as a potential conflict of interest.
